# Imaging Sodium Dendrite Growth in All‐Solid‐State Sodium Batteries Using ^23^Na *T*_2_‐Weighted Magnetic Resonance Imaging

**DOI:** 10.1002/ange.202013066

**Published:** 2020-11-24

**Authors:** Gregory J. Rees, Dominic Spencer Jolly, Ziyang Ning, T. James Marrow, Galina E. Pavlovskaya, Peter G. Bruce

**Affiliations:** ^1^ Department of Materials University of Oxford Parks Road Oxford OX1 3PH UK; ^2^ Department of Chemistry University of Oxford South Parks Road Oxford OX1 3QZ UK; ^3^ The Henry Royce Institute Parks Road Oxford OX1 3PH UK; ^4^ The Faraday Institution Harwell Campus Didcot OX11 0RA UK; ^5^ Sir Peter Mansfield Imaging Centre School of Medicine University of Nottingham Nottingham NG7 2RD UK; ^6^ NIHR Nottingham Biomedical Research Centre University of Nottingham Nottingham NG7 2RD UK

**Keywords:** all-solid-state electrolytes, batteries, magnetic resonance imaging, NMR spectroscopy, sodium

## Abstract

Two‐dimensional, Knight‐shifted, *T*_2_‐contrasted ^23^Na magnetic resonance imaging (MRI) of an all‐solid‐state cell with a Na electrode and a ceramic electrolyte is employed to directly observe Na microstructural growth. A spalling dendritic morphology is observed and confirmed by more conventional post‐mortem analysis; X‐ray tomography and scanning electron microscopy. A significantly larger ^23^Na *T*_2_ for the dendritic growth, compared with the bulk metal electrode, is attributed to increased sodium ion mobility in the dendrite. ^23^Na *T*_2_‐contrast MRI of metallic sodium offers a clear, routine method for observing and isolating microstructural growths and can supplement the current suite of techniques utilised to analyse dendritic growth in all‐solid‐state cells.

## Graphical abstract

Knight‐shift *T*_2_‐weighted MRI measurements of dendrite formation in all‐solid‐state batteries show direct contrast between the bulk electrode and the dendrite formation. This significantly increased *T*_2_ of the spalling dendrite formation is attributed to a local motion of the metallic Na ions.

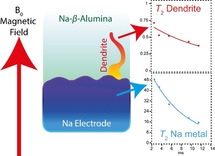

All‐solid‐state batteries (ASSB) with a ceramic electrolyte and an alkali metal anode could deliver a step‐change in energy storage and safety. The use of solid‐state electrolytes has numerous advantages over the conventional organic electrolytes such as the ability to use metal anodes, removal of volatile and flammable electrolyte organics, and they open up the possibility of Li‐Air and Li‐Sulphur cathodes (which have higher volumetric density).^[1]^ These advantages are protracted when coupled with sodium anodes which allow the use of aluminium current collectors (whereas more‐expensive Cu is required for Li), sodium also has a significantly higher natural abundance (2.36 % abundance in the earth's crust) compared to that of conventionally used lithium (<0.002 %) and, therefore, offers more security against a volatile Li market.[^1^, ^2^] One of the greatest barriers to the progress of ASSBs is the formation of dendrites (filaments of alkali metal) on charging that penetrate the ceramic leading to a short‐circuit and cell failure.^[3]^ Dendritic growths in ASSB systems have different morphologies to their solution counterparts, which can be correlated to the electrochemistry. These growths have been categorized into four discrete morphologies; straight, branching, spalling, and diffuse.[^4^, ^5^]

Imaging such dendrites is essential to understand their growth and to develop mechanisms to prevent their formation. Magnetic resonance imaging (MRI) can provide non‐destructive, isotope specific, structural, time‐resolved, and quantifiable multi‐dimensional information.^[6]^ Both ^1^H and ^7^Li MRI and magnetic resonance spectroscopy (MRS) have been utilised to probe dendrites in liquid electrolyte electrochemical cells,^[7]^ and ^7^Li chemical shift imaging has explored Li microstructural growth in ASSBs.^[8]^ Recent work by Bray et al. shows that *in‐operando* ^23^Na MRI and MRS studies on sodium cells with organic liquid electrolytes were able to determine the sodium speciation upon galvanostatic cycling.^[9]^ *In‐situ* ^23^Na nuclear magnetic resonance (NMR) during electro‐deposition of Na, shows that reversible high‐surface‐area mossy and/or dendritic structures can be observed and attributed to a nucleation mechanism.^[12]^ Direct *T*_2_ contrast MRI on any battery material (^1^H, ^6/7^Li and ^23^Na) has never been investigated. However, NMR relaxometry measurements (spin‐lattice relaxation; *T*_1_, spin‐spin relaxation; *T*_2_) have been vital in understanding ion dynamics in a range of battery systems.^[13]^

Imaging solid‐state electrolytes is technically more difficult than their solution counterparts as the NMR linewidths are substantially broader, this causes the *T*_2_/*T*_2_^*^ to be short and hence the signal to dephase during the application of the imaging gradients. Conventional ^1^H MRI is not possible in ASSBs due to the lack of protons in the system and if the system did contain protonated groups these would have broad intrinsic linewidths (kHz) which would dephase during the application of gradients. Therefore, work in this field has focussed on the narrower linewidths of ^7^Li and its distribution within the solid‐electrolyte.^[14]^ Likewise, ^23^Na MRI is considered more challenging than ^7^Li, as it has a significant chemical shift, greater quadrupole moment, and lower sensitivity than ^7^Li.^[15]^ However, these larger properties can be utilised to achieve greater image contrast. Specifically, with a time‐incremented series of MRI experiments, spin‐spin (*T*_2_) relaxation can be measured at each spatial element of the object and an MRI image based on relaxation rather than spin density can be produced. The achieved spatial contrast supplies greater information to the images and allows one to comment on the dynamics or local site symmetry of individual pixels. Here we report for the first time, *T*_2_ contrast ^23^Na MR images of metallic Na electrodes in a pristine state and after short‐circuiting. The *T*_2_ maps of the electrochemical cells allow us to comment on the Na‐ion dynamics of the formed features. As ^23^Na is significantly more difficult than ^7^Li, this methodology can readily be adapted to more conventional Li‐ASSBs and liquid electrolyte systems.

Two‐dimensional spin‐echo Knight‐shift ^23^Na MRI images of the symmetrical all‐solid‐state Na | Na‐*β*′′‐Alumina | Na cell are shown for the pristine cell in Figure 1 c and a cell after passing a current in Figure 1 d, with the dendrite apparent in the latter. A thorough description of the experimental setup is given in the SI (Figure S1). These intensity images show that the amount of signal originating from the dendritic feature is limited due to the deficiency of ^23^Na nuclear spins in this structure, despite this, the dendrite is still observable in Figure 1 d. Herein lies the major obstacle with materials MRI; to increase the resolution (or to produce 3D representations) a smaller area of space is needed to be sampled, however, this means that the number of nuclear spins contributing to the signal, in this given area, is reduced and, therefore, the experimental times increase dramatically. MRI has circumvented this issue by employing contrast driven sequences which can isolate the area of interest and, thus, does not require such high‐resolution images.

**Figure 1:**
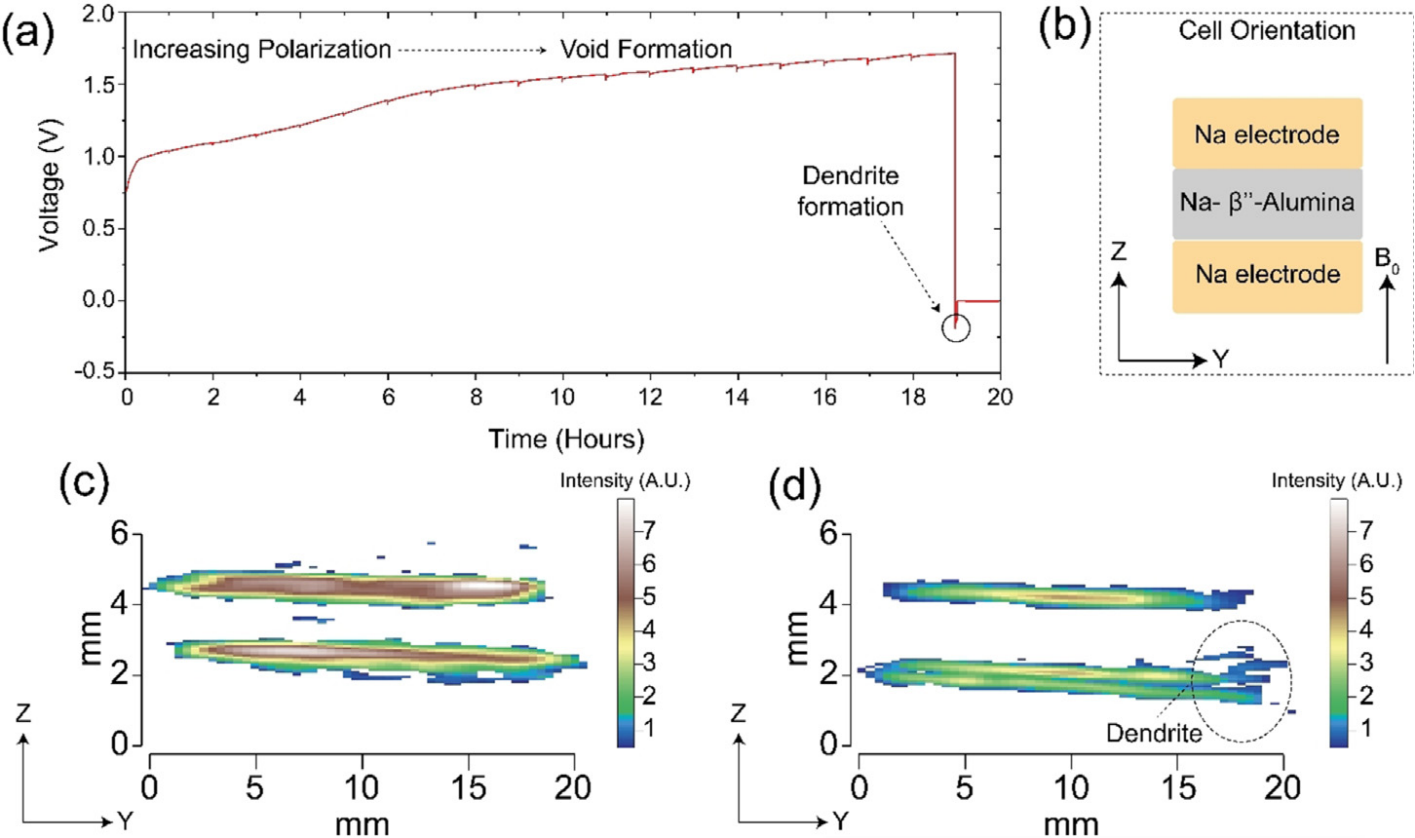
a) Galvanostatic cycling of a Na | Na‐*β*′′‐Alumina | Na cell under 1 MPa stack‐pressure and a current density 0.5 mA cm^−2^. A charge of 9.5 mAh cm^−2^ is passed on the first 1/2 cycle during which increased polarisation is observed, consistent with the formation of voids at the electrode as Na is stripped, as shown previously.^[10]^ On current reversal, the cell short‐circuits rapidly consistent with the formation of dendrites. b) The arrangement of the cell (imaged in the *z*,*y* plane) within the magnetic field (*B*_0_), this orientation is conserved throughout all the images. The traditional, intensity, Knight shift ^23^Na MRI images of the c) pristine cell and d) cell after short‐circuit containing a dendritic growth (highlighted) at the edge of the cell, which is consistent with ion migration along the electric field lines.^[11]^ The equivalent gradient‐echo intensity images are given in Figure S4.

The microstructural growth observed in Figure 1 d follows a spalling morphology previously observed by Kazyak and co‐workers, other dendritic morphologies which form in ASSBs are also discussed.^[4]^ A spalling morphology is formed when a dendritic crack propagates back to the surface, forming a conical surface fracture which often precedes the dendrite fully traversing the solid‐electrolyte and causing a short circuit. Although the dendrite must have extended from counter to working electrode at the moment of short‐circuit (voltage=0 V in Figure 1 a), at the time of imaging there remains no observable dendrite transversing the electrodes and only the spallation section of the dendrite is observed. This is due to the high current passing through the thinnest parts of the dendrite causing Joule heating, which burns away the thinnest sections of the dendrite. Growth of a metallic Na dendrite parallel to the magnetic field causes the NMR signal to shift to a higher frequency by ≈5 ppm (from 1126 up to 1131 ppm),[^9^, ^12^] this is due to orientation dependant nature of the Knight shift.^[16]^ The shift is smaller than that observed in ^7^Li as Na has a reduced bulk magnetic susceptibility.^[2]^ The spalling nature of the microstructural growth seen here has no distinct directionality, with respect to the magnetic field (*B*_0_, Figure 1 b), therefore no obvious secondary peak formation is observed in the MRS spectra (Figure S2b).

The skin depth of sodium is 11 μm at 105 MHz (9.4 T), therefore, only the top and bottom 11 μm of metallic Na is observed in 2D projections. At 9.4 T the lithium‐7 skin depth is comparable, ≈12 μm. As dendrites are narrow thin filaments of metallic species, their formation should increase the number of ^23^Na nuclei visible in the MRS and MRI experiments as more nuclei are moved into areas below the skin‐depth and are, therefore, accessible to the radio‐frequency pulse. There are two possible reasons for not notably detecting an increase in ^23^Na signal, the first is that large spherical spalling morphology of the dendritic growth does not reduce the skin depth and secondly the size of the parallel component of the dendrite may be below the limit of detection. The lack of chemical shift change and/or increase in signal intensity means that conventional ^23^Na NMR cannot be utilized to observe dendritic growth in these ASSBs.

More established *ex‐situ* post‐mortem dendrite characterisation techniques, X‐ray computed tomography and scanning electron microscopy (SEM), were completed to confirm the nature of the dendrite's morphology. The X‐ray computed tomography image for the pristine cell is shown in Figure 2 a and the cell after shorting is given in Figure 2 b. A spalling morphology dendritic formation is observed in the shorted cell, which is in good agreement with the ^23^Na MRI. The pixel size of the tomography images is 4.66 μm and no clear evidence of a dendrite or crack is observed within the electrolyte. Likewise, the lack of dendrite observed penetrating the length of the electrolyte agrees with the MRI results and suggests that Joule heating has burnt away the dendrite. The corresponding SEM images are presented in Figure 2 c and an increased resolution image focusing on part of the spalling feature is given in Figure 2 d, with the corresponding EDX images in Figure 2 e. The post‐mortem cross‐sectional images show the formation of a spallation crack. This crack is filled with a metallic material assumed to be Na, however, this was not confirmed with certainty as although there is a weak Na signal from the dendritic area, the response is dominated by a strong C signal, likely due to the reaction of adventitious carbon species with the highly reactive and newly exposed Na surfaces.

**Figure 2:**
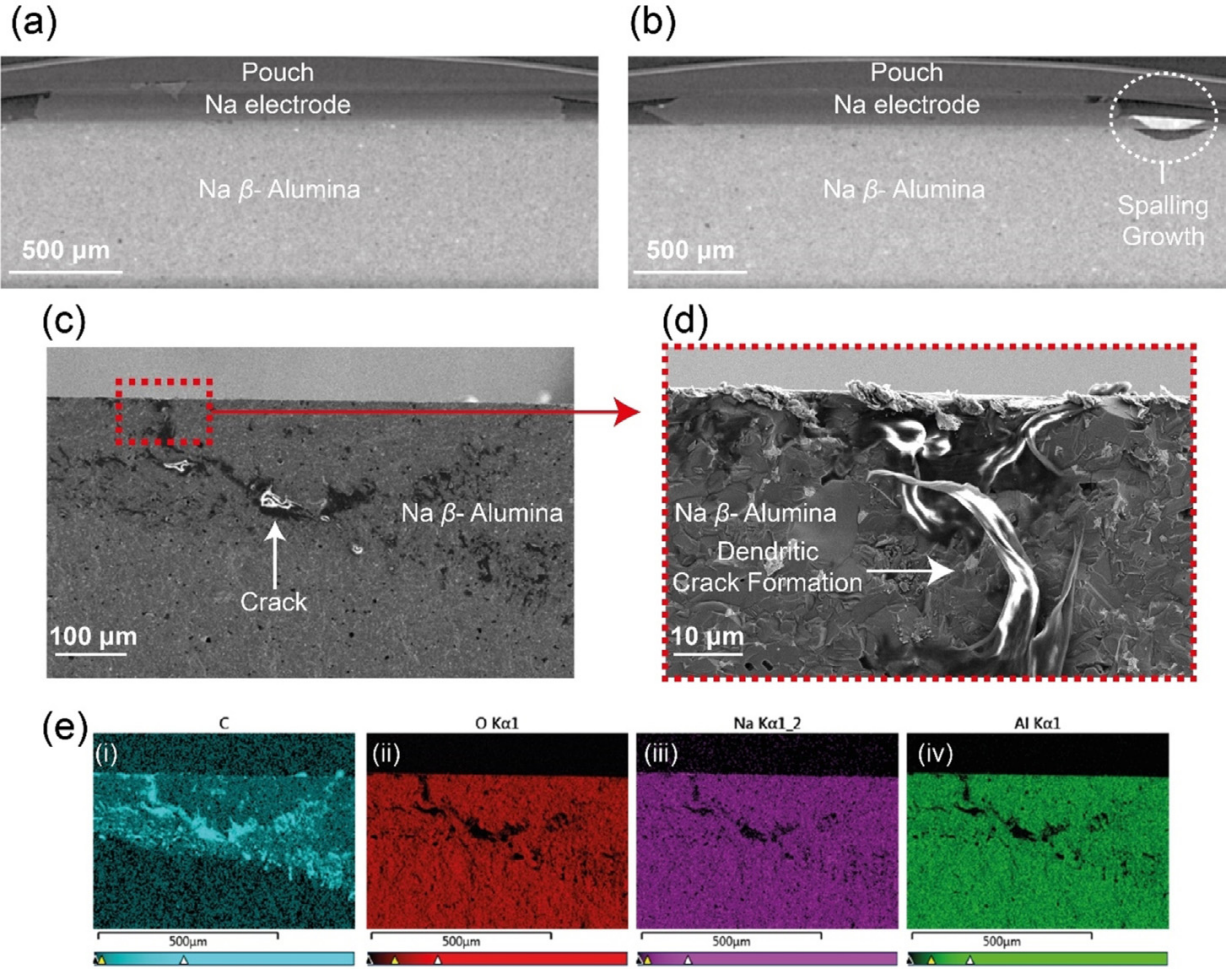
The X‐ray tomography images (4.66 μm resolution) of the a) pristine cell and b) cell after short circuit showing the spalling morphology. Scanning electron micrographs illustrating the dendritic crack formation with increasing resolution, c) 100 and d) 10 μm scale. e) The corresponding energy‐dispersive X‐ray (EDX) images highlighting the distributions of (i) C, (ii) O, (iii) Na, and (iv) Al.

These images highlight the difficulties with definitively imaging dendrites in ASSBs. The nano‐scale of the crack and dendrite makes visualizing these formations challenging by X‐ray tomography and although higher resolution can be achieved, these are not routinely accessible for post‐mortem cell‐failure analysis or industrial use. In addition, SEM/EDX is often imperfect as its cross‐sectional nature makes it a destructive technique, only able to show a 2D area of a 3D dendrite and EDX is often not able to achieve the desired specificity of chemical information.

The effect of *T*_2_ weighting the MR images is shown in Figure 3 a for the pristine and Figure 3 c for the cell after short‐circuit, with their respective *T*_2_ histograms given in Figure 3 b and d. With current experimental parameters, a full *T*_2_ map (5 echo increments) can be achieved in ≈11.5 hours, using a spin‐echo acquisition scheme. Although this timescale is too long for *in‐situ* or *in‐operando* measurements of dendritic growth, making this method only applicable for post‐mortem analysis, one needs to recall that these measurements were completed on commercially available equipment (suitable for medical and biological applications) and optimisation of the coil size, probe power handling, gradient strength, and magnetic field strength will all significantly reduce the experiment time. The superior contrast of the dendrite (Figure 3 c, red region) is due to a longer *T*_2_ of the Na nuclei in the growth, this is an isolated peak in the corresponding distribution histogram (Figure 3 d; labelled, yellow peak).

**Figure 3:**
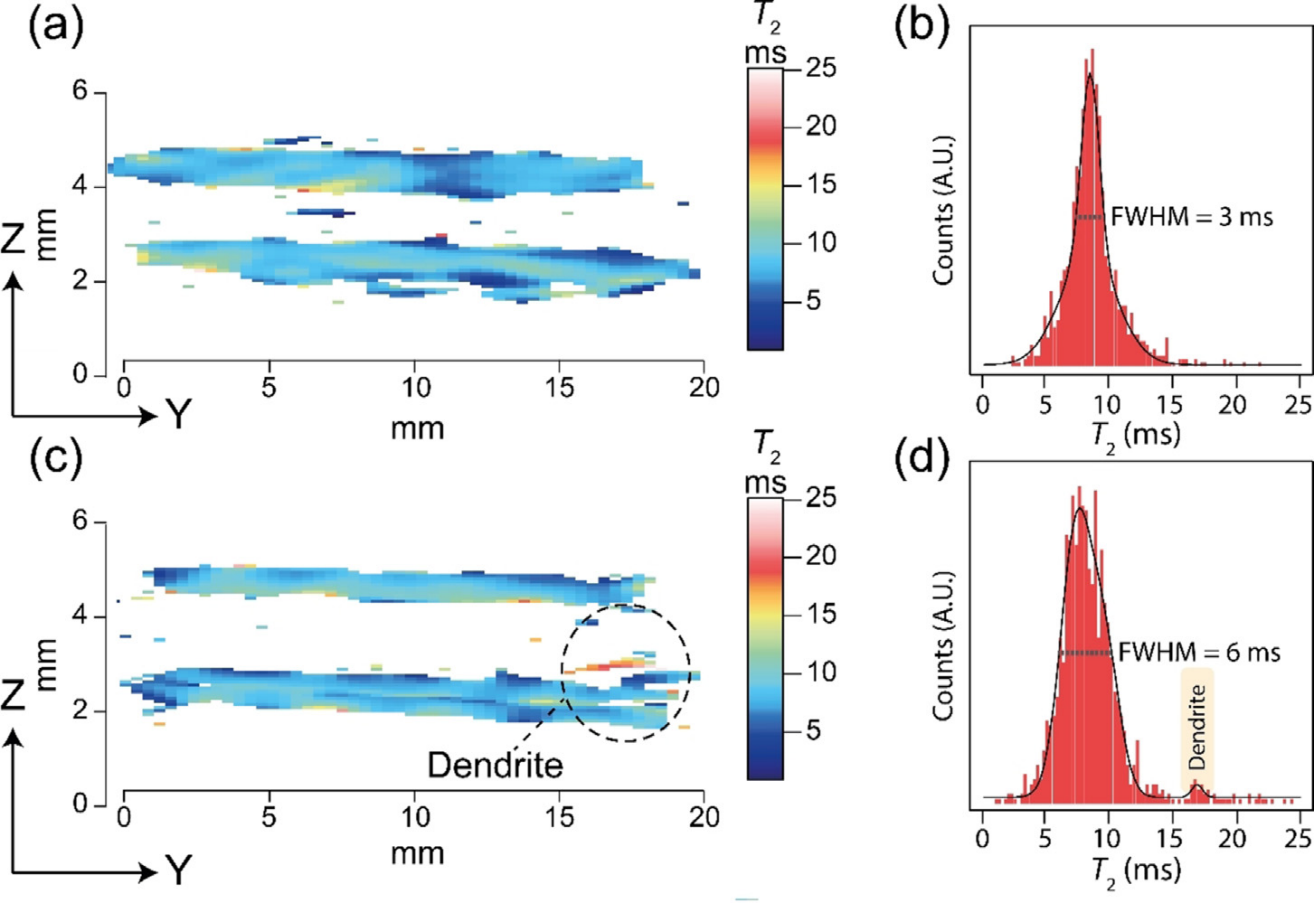
The *T*_2_ weighted contrast maps (with the same orientation and scale as Figure 1 c and d) of a) the pristine symmetrical cell and c) the cell after short‐circuit. The dendrite is highlighted and has a significantly increased *T*_2_. The respective total *T*_2_ distributions are given in the histograms (b) and (d), with the isolated dendrite distribution highlighted. The individual ^23^Na images which were used to produce the *T*_2_ weighted maps are given in Figure S3.

The *T*_2_ exponential decay curves for a range of pixels in the bulk electrode (Figure 4 c) and the dendrite (Figure 4 a), with respect to the background noise (Figure 4; given in dark blue), illustrates the appreciable differences between the *T*_2_ relaxation characteristics of the dendrite and the bulk metal electrodes. The noise level in these images is minor even at extended echo delays. In the dendrite pixels, a signal to noise ratio of ≈4 is achieved despite the lack of ^23^Na nuclei in these sites. The *T*_2_ relaxation of the electrodes varies from 5–10 ms in both the pristine and cell after short‐circuiting, with an increased full width at half maximum height (FWHM, Figure 3 b,d) being observed in the cell after short‐circuiting. This FWHM increase is attributed to increased local disorder of the ^23^Na nuclei due to the formation of voids in the electrode.[^10^, ^17^] The *T*_2_ relaxation times in the dendritic growth are significantly longer (>12 ms) with a cluster of *T*_2_’s at 16 ms (Figure 4 b).

**Figure 4:**
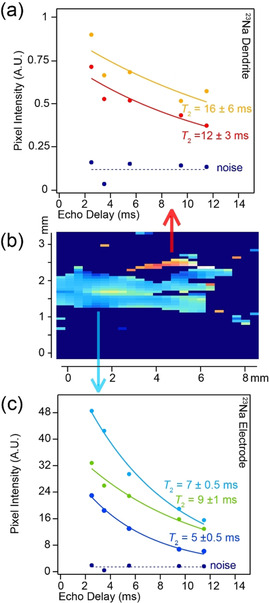
The spin‐echo decay curves showing the determined *T*_2_ parameters for a range of pixels from a) the dendritic growth and c) the bulk electrode. The curves are colour coded to the pixels shown in the expanded 2D *T*_2_‐contrast image presented in (b). Each pixel intensity normalised curve is fitted to a mono‐exponential with a constant offset determined by the noise level (given in dark blue).

Spin‐spin (*T*_2_) relaxation is a time constant which describes the signal decay in the transverse (*x,y*) plane and is routinely utilized in conventional *in‐vivo* medical ^1^H MRI to generate image contrast.^[18]^ The *T*_2_ relaxation mechanism, in solids, is attributed to numerous factors such as dipole‐dipole coupling, anisotropies, quadrupole effects, and local motion. The dominant *T*_2_ relaxation effect in metals is Pauli paramagnetism coupled with strong dipole‐dipole coupling.^[19]^ The 100 % natural abundance ^23^Na spins directly couple through their nuclear magnetic moments, and indirectly through the intermediary of the conduction electron spins, the so‐called pseudo‐dipolar and pseudo‐exchange coupling, respectively.^[19]^ The *T*_2_ weighted map of the cell after short‐circuit shows significant contrast between the Na electrodes (*T*_2_ span=5–12 ms) and the increased relaxation of the dendrite (*T*_2_ span=16–17 ms). As the dendrite growth has reduced dimensions with reduced local symmetry, compared to the electrode, then one would expect the *T*_2_ to be shorter than the bulk Na metal electrode, due to increased quadrupolar/anisotropic effects. In metallic NMR, the signals are broadened when particle sizes are lower than 10 nm.^[20]^ Below 10 nm the local symmetry experienced by the ^23^Na nuclei will distort away from cubo‐octahedral, causing systematic linewidth broadening as the particle size decreases. This distortion of the local symmetry will also affect the difference between the highest occupied molecular orbital (HOMO) and the lowest unoccupied molecular orbital (LUMO), resulting in a change in the Knight shift.^[21]^ As there is no observed shift or broadening in the ^23^Na peak position from either the electrode and the dendrite (Figure S2b), then it may be assumed the dendrite is larger than 10 nm. Incidentally, tomography images were collected with a 4.66 μm pixel size and no dendrite was observed in these images. Therefore, we propose that the dendrite width ranges from tens to hundreds of nanometres. The spalling microstructural growth observed in the ^23^Na images is 23–46 μm in size (one—two pixels along the *z*‐axis).

The absence of broadened features in the NMR suggests that the increased spin‐spin relaxation must be dominated by another effect. We attribute the substantially increased *T*_2_ to increased mobility of the ^23^Na nuclei in the dendritic filament, this mobility would reduce the dipole‐dipole coupling and, hence, increase the spin‐spin relaxation. The mechanisms governing self‐diffusion in locally disordered materials are driven by their defects. Point defects, such as vacancies or interstitials, give increased cation self‐diffusion.^[22]^ Dislocations, grain boundaries, phase boundaries, and free surfaces are other types of defects found in solids cause significant clusters of local defects and act as a diffusion barrier.^[23]^ Therefore, the observed increase in *T*_2_ within the dendrite could be due to a higher concentration of point defects or a lower concentration of dislocations, grain boundaries, phase boundaries or free surfaces within the dendrite compared to the bulk metal electrode, likely due to the unique conditions of plating Na at a fast rate into a thin crack with a small aperture.

Regardless of the mechanism, the effect of the different *T_2_* values between the bulk electrode and dendrite is the ability to resolve one from another and to discretely determine where a dendrite begins and the electrode stops. The isolation of the minor peak at a *T*_2_ of ≈16 ms (Figure 3 d) of the cell after short‐circuit shows that the ^23^Na nuclei in the dendrite is in a significantly different dynamic environment to the bulk metal in the electrode, and offers the opportunity to develop *T*_2_ resolved experiments to solely image the dendrite. A broader *T*_2_ distribution (Figure 3 d) in the electrode region (5–12 ms) of the shorted cell is evident and can be attributed to a greater range of local Na site symmetries and the formation of voids.

The application of *T*_2_ weighted ^23^Na MRI is a promising technique to directly observe the formation and determine the structural dynamics of dendrites in ASSBs. The drive for contrast imaging is to remove resolution limits and here, despite the dendrite being smaller than the resolution of both ^23^Na MRI and tomography, one can still observe the dendrite by MRI. The *T*_2_ of the dendrite is significantly longer than the *T*_2_ of the bulk metal electrode. Due to a lack of broadening observed in the corresponding NMR spectra, the longer *T*_2_ must be attributed to increased Na dynamics in the dendrite. This contrast driven methodology can also be utilized on liquid electrolyte cells, which have different dendritic morphologies to ASSBs.^[24]^ One limitation of ^23^Na MRI is the inability to image the crack development in ASSBs, therefore we recommend a multimodal imaging approach, combining X‐ray computed tomography to track morphological changes in the cells, high‐resolution elemental content can be achieved from *ex‐situ* SEM, and dynamic information can be attained from contrast‐driven MRI. This multimodal approach allows one to image crack formation, microstructural growth, ion‐dynamics, and any dendritic formations.^[25]^

## Conflict of interest

The authors declare no conflict of interest.

## Supporting information

As a service to our authors and readers, this journal provides supporting information supplied by the authors. Such materials are peer reviewed and may be re‐organized for online delivery, but are not copy‐edited or typeset. Technical support issues arising from supporting information (other than missing files) should be addressed to the authors.

Supplementary
