# Supplementary material for: Imaging Sodium Dendrite Growth in All‐Solid‐State Sodium Batteries Using 23Na T 2‐Weighted Magnetic Resonance Imaging
Source: Angew Chem Weinheim Bergstr Ger. 2020 Nov 24;133(4):2138–43. doi: 10.1002/ange.202013066 (PMC10946918; doi:10.1002/ange.202013066)
Supplement: Supplementary file 1 — Supplementary [file ANGE-133-2138-s001.pdf]

Angewandte Chemie
Eine Zeitschrift der Gesellschaft Deutscher Chemiker

Supporting Information

Imaging Sodium Dendrite Growth in All-Solid-State Sodium Batteries Using ^{23}Na T_2-Weighted Magnetic Resonance Imaging

Gregory J. Rees, Dominic Spencer Jolly, Ziyang Ning, T. James Marrow, Galina E. Pavlovskaya, and Peter G. Bruce**

ange_202013066_sm_miscellaneous_information.pdf

Contents

Page 2; **Experimental Procedures**

Page 4; **Figure S1 –** Experimental Setup and Cell Orientations.

Page 5; **Figure S2 –** ^{23}Na MRS and Relaxometry Measurements.

Page 6: **Figure S3 –** ^{23}Na MRI Spin-Echo Images with Variable *τ* Increments.

Page 7: **Figure S4 –** Proof of Principle Gradient Echo ^{23}Na MRI images.

Experimental Procedures

Magnetic Resonance Imaging. All ^{23}Na ($I = {}^3/_2$) were completed at 9.4 T (υ_0 = 105.98 MHz) using a Bruker Advance III HD spectrometer. Spin-echo 128 x 32 images with $25 \, x \, 8$ mm^2 FOVs were collected with 256 transients, a 1s recycle delay, and 5 echo delays incremented up to 24 ms. The raw data were zero-filled to 256 x 64 and apodised in each dimension using sinbell squared function before image reconstruction. T_2 maps were obtained by non-linear least-squares fitting of the ^{23}Na intensity in each voxel, as a function of the echo delay, to a mono-exponential function using Prospa 3.12 (Magritek, Germany). A commercially available wide-bore Bruker 30 mm ^{23}Na microimaging coil was used for all measurements. An excitation bandwidth of 2.2 kHz (${}^{\pi}/_2$) was utilized for all experiments, this pulse width is acceptable for the Knight-shifted resonance, which has an FWHM of ~130 Hz. The limited power handling capability of commercially available microimaging coils of this diameter and the use of longer pulses is advantageous in this case as it does not excite the non-metallic region of the sodium spectrum leading to sodium images free of chemical shift artefacts. The use of widely available MRI equipment maximises the applicability of the study and specifically designed MRI coils and pulse sequences would limit the access to these observations. The *z*-axis and the cell were aligned with the B_0 of the magnetic field, and all images are presented in the *z,y* plane (schematic given in Fig. 1b). The corresponding proof of principle gradient-echo intensity images and their experimental parameters are presented in Figure S2.

The bulk T_2 MRS measurements (Fig. S1c) were performed on sodium metal (Fig. S1c, bulk metal) and a shorted cell with both electrodes etched away, leaving only the ceramic electrolyte and the embedded dendrite (Fig. S1c, dendrite).

The samples were packaged in an argon sealed pouch for all experiments, and no signs of sodium metal oxidation were present during the experiments or 10 days after experimentation, suggesting the packing and stability of the samples under experimentation was effective over a long period (10 days, as shown in Fig. S1b).

Preparation of Na | Na-*β”*-Alumina | Na cells: Discs of Na-*β”*-Alumina measuring 20 mm in diameter and 2 mm thick were purchased from Ionotec and their surfaces prepared by polishing and heat-treatment as described previously.[1] Na- *β”*-Alumina was chosen for this study as it is a realistic candidate for Na anode SSBs. It has a very high ionic conductivity of 2 mS cm^{-1}, is commercially available, and is very well characterised in the literature. Na metal electrodes, 18 mm in diameter, were cut from Na ingot and after having any oxide layer carefully removed with a scalpel, were pressed against the ceramic electrolyte in an argon-filled glovebox. The symmetric cell was then sealed within a pouch cell utilising aluminium current collectors.

The cell size and characteristics are archetypical for studying dendrite formation mechanisms in ASSBs, which allows plating and stripping behaviour simultaneously on either side of the cell and removes any unnecessary cathode complexity. When the spatial resolution of a technique is limited (such as in neutron imaging), it is useful to use cells which are as large as possible, therefore, when imaging the whole sample one can achieve a better relative spatial resolution. For this is the reason larger cells are typically used to study dendritic growth processes in ASSBs.

Galvanostatic cycling of Na | Na-*β”*-Alumina | Na: Cycling was conducted under a stack-pressure of 1 MPa. The cell’s working electrode was stripped at a current density of 0.5 mA cm^{-2} moving capacity of 9.5 mAh cm^{-2} in 19 steps of 0.5 mAh cm^{-2} charge passed. These conditions were chosen to create significant contact loss at the working electrode / solid electrolyte interface[1] which is evident by the approximately 1 V increase in polarisation over

the course of the stripping (Fig. 1a) and by the voids observed in the interpolated Knight shift ^{23}Na MRI image (Fig. 1d). Upon reversing the current and plating the working electrode, the voltage was observed to drop almost immediately to 0 V, indicating a short-circuit due to dendrite growth.

Scanning Electron Microscopy (SEM) and X-ray Computed Tomography: The pristine and short-circuited Na | Na-*β''*-Alumina | Na cells were sealed into pouches after the current collectors were removed in an argon-filled glovebox and scanned inside a Zeiss Xradia Versa 510 X-ray microscope. For each tomogram, 3201 equiangularly distributed projections were taken over 360^0, with a beam energy of 120 keV and a resulting voxel size of 4.66 µm. The projections were processed and reconstructed into tomograms using the Zeiss Scout-and-Scan Reconstructor software. Post-processing of the tomograms was completed using ImageJ for cropping and reslicing.[2]

The short-circuited cell was then disassembled in an argon environment and the Na electrode were removed. The ceramic was then cross sectioned across a spalling feature, located by XCT, with the ultrasonic cutter with a tungsten carbide blade (SONOTEC). The cross-sectioned cell was transferred into a Zeiss Merlin scanning electron microscope using an airtight transfer device (Gatan) for SEM and EDX analysis. Secondary electron images were collected using a probe current of 200 pA and an acceleration voltage of 5 kV.

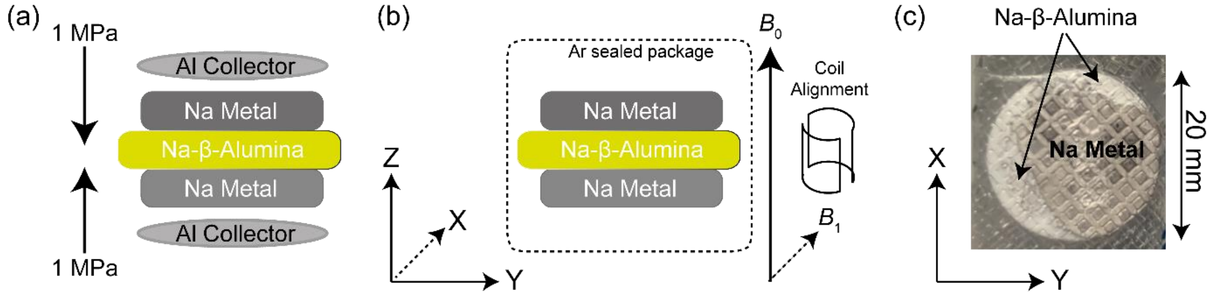

Figure S1: (a) A schematic showing the experimental setup and pressure of the cell used for galvanostatic cycling, as outlined in the experimental section. (b) The orientation of the cell with respect to the magnetic field (B_0), the pulse field (B_1) and the coil. (c) A top view (x,y plane with respect to B_0) of the cell showing the Na metal electrode and the underlying Na-β-alumina ceramic electrolyte.

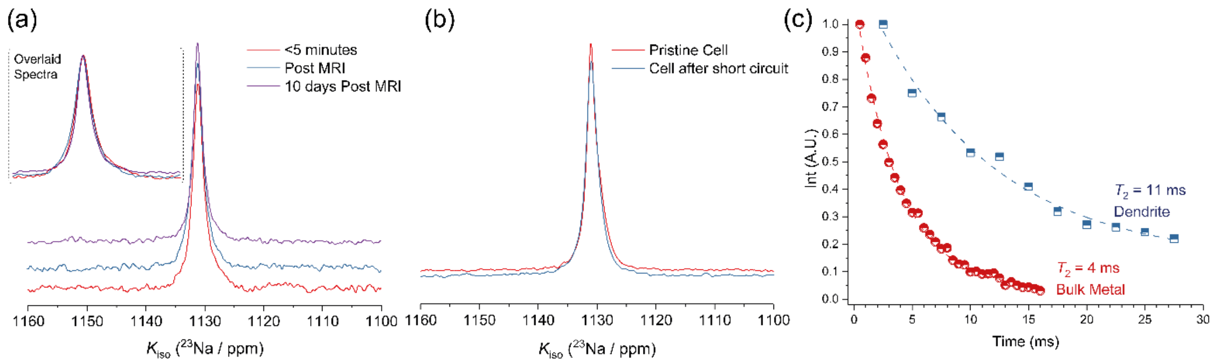

Figure S2: (a) The Knight-shifted MRS spectra of the pristine and short circuit symmetrical cells. These spectra were achieved on the post short-circuit cell and were taken less than 5 minutes after cycling (red), after the MRI experiments (blue), and 10 days after the MRI experiments (purple), with the inset showing the three spectra overlaid. The lack of any discernible differences suggests the cell was not exposed to any oxygen/water and the MRI experiment did not affect the structure (*i.e.* localised heating effects). (b) Overlay of the Knight-shifted MRS spectra of the symmetrical cell in the pristine state and after a short circuit, showing no obvious differences, this suggests that conventional NMR cannot be used to observe the dendrite. (c) The spin-echo bulk T_2 exponential decays, measured by MRS, of the bulk ^{23}Na metal (4 ms) and an isolated dendrite (11 ms). The possible deviations in the T_2 between the MRS and MRI data (Fig. 4a and c) are attributed to the 1 MPa pressure applied to the MRI cycled cell. The dendrite was isolated in the ceramic by polishing the metallic electrodes from a shorted cell.

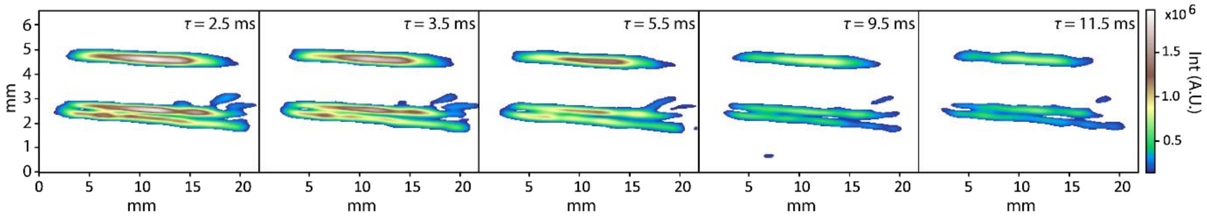

Figure S3: The 2D Knight-shifted ^{23}Na MRI at *τ* increments varying from 2.5 to 11.5 ms for the cell after short circuit (zero-filled to 256 x 256 points). The analogous 128 x 32 images were used to generate the T_2 weighted image given in Fig. 3c.

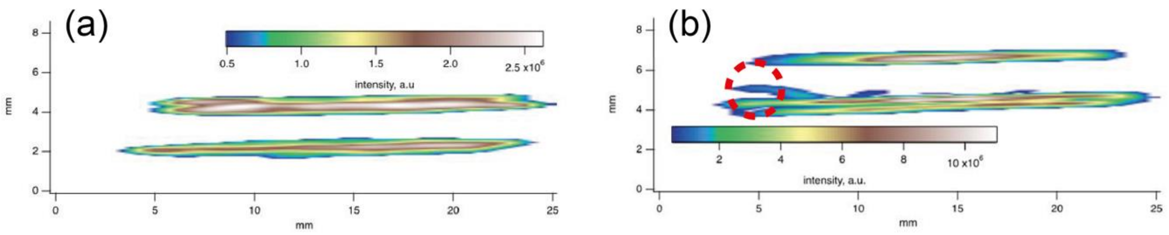

Figure S4: The gradient-echo intensity 128 x 32 images of (a) the pristine cell and (b) the cell after short circuit. The dashed red circle highlights the spallation feature (the images are presented in the $-z,y$ projections, compared to the corresponding spin-echo images in Fig. 1). The images have $8\ x\ 25$ mm^2 FOVs, were collected with 512 transients, and 1s recycle delay with a gradient refocusing time of 590 ms. The raw data were zero-filled to 256 x 64 and apodised in each dimension using a sinbell squared function before image reconstruction. The long 1s delay was chosen to alleviate any potential thermal effects. As the imaging was not performed *in-situ* and rather for the proof of principle the recycle delay was not adjusted to $5T_1$ of ^{23}Na in the metallic phase (T_1 of bulk ^{23}Na metal <100 ms).[3] In principle, one could reduce the imaging time using this methodology to <20 minutes by using an appropriate recycle delay.

Notes

Supporting research data has been deposited in the Oxford Research Archive and is available under this DOI: 10.5287/bodleian:1amymAzYZ

References

[1] D. Spencer Jolly, Z. Ning, J. E. Darnbrough, J. Kasemchainan, G. O. Hartley, P. Adamson, D. E. J. Armstrong, J. Marrow, P. G. Bruce, *ACS Applied Materials & Interfaces* **2020**, *12*, 678-685.

[2] C. T. Rueden, J. Schindelin, M. C. Hiner, B. E. DeZonia, A. E. Walter, E. T. Arena, K. W. Eliceiri, *BMC Bioinformatics* **2017**, *18*, 529.

[3] P. M. Bayley, N. M. Trease, C. P. Grey, *Journal of the American Chemical Society* **2016**, *138*, 1955-1961.
